# Treatment of Retinoblastoma: What Is the Latest and What Is the Future

**DOI:** 10.3389/fonc.2022.822330

**Published:** 2022-04-01

**Authors:** Paula Schaiquevich, Jasmine H. Francis, María Belén Cancela, Angel Montero Carcaboso, Guillermo L. Chantada, David H. Abramson

**Affiliations:** ^1^Unit of Innovative Treatments, Hospital de Pediatría JP Garrahan, Buenos Aires, Argentina; ^2^National Scientific and Technological Research Council (CONICET), Buenos Aires, Argentina; ^3^Ophthalmic Oncology Service, Memorial Sloan Kettering Cancer Center, New York, NY, United States; ^4^Department of Ophthalmology, Weill/Cornell Medical School, New York, NY, United States; ^5^Hemato-Oncology, Hospital Sant Joan de Déu, Barcelona, Spain; ^6^Institut de Recerca Sant Joan de Déu, Barcelona, Spain; ^7^Institute for Translational Research, Universidad Austral, Buenos Aires, Argentina; ^8^Research Department, Fundacion Perez-Scremini, Montevideo, Uruguay

**Keywords:** innovative treatments, intra-arterial chemotherapy, intravitreal injections, retinoblastoma, pharmacology, chemotherapy, adenovirus

## Abstract

The management of retinoblastoma, the most common intraocular malignancy in children, has changed drastically over the last decade. Landmark developments in local drug delivery, namely, safer techniques for intravitreal chemotherapy injection and ophthalmic artery chemosurgery, have resulted in eye globe salvages that were not previously attainable using systemic chemotherapy or external beam irradiation. Novel drugs, oncolytic viruses, and immunotherapy are promising approaches in the treatment of intraocular retinoblastoma. Importantly, emerging studies of the pattern of tumor dissemination and local drug delivery may provide the first steps toward new treatments for metastatic disease. Here, we review recent advances in retinoblastoma treatment, especially with regard to local drug delivery, that have enabled successful conservative management of intraocular retinoblastoma. We also review emerging data from preclinical and clinical studies on innovative approaches that promise to lead to further improvement in outcomes, namely, the mechanisms and potential uses of new and repurposed drugs and non-chemotherapy treatments, and discuss future directions for therapeutic development.

## Introduction

Retinoblastoma is a highly curable neoplasm in high-income countries, where patient survival exceeds 99%, making it the most curable of all pediatric cancers. In striking contrast, in many lower-income countries, most patients present with disseminated and metastatic disease, which is almost always fatal (1, 2).

In high-income countries, eye-sparing treatments have been used for decades. In the late 1990s there was an evolution in conservative treatment from local, eye-directed therapies such as external beam radiotherapy (EBRT) toward systemic chemotherapy combined with aggressive focal therapies. This change in approach aimed to reduce the use of EBRT, which has been consistently associated with a higher risk of second malignancies (3). The use of systemic chemoreduction was effective for tumor control in eyes with less advanced tumors, avoiding the use of EBRT (4, 5). However, the majority of eyes worldwide (75%) present with more advanced tumors with massive vitreous or subretinal seeding and retinal detachment, where both radiation and systemic chemotherapy can rarely save the eye or vision (6). Although this treatment results in reduced long-term toxicity by avoiding EBRT in many patients, its acute toxicity, though manageable in developed countries can be fatal in up to 4–5% of patients in less resourced settings (7). Moreover, most studies showed an additive risk for secondary malignancies when chemotherapy is combined with EBRT (8). Ototoxicity caused by carboplatin and cases of fatal chemotherapy-induced leukemia were also reported in non-irradiated children with retinoblastoma (9–12). So, from the mid-2000s on, most groups moved away from systemic chemotherapy to advanced techniques for more selective ocular delivery to increase drug exposure in the tumor, maximizing efficacy and minimizing the probability of adverse events.

The introduction of ophthalmic artery chemosurgery (OAC) led to remarkable success in treating eyes with more advanced disease in which systemic chemotherapy had poorer results (13–20). In the 2010s intravitreous chemotherapy (IVi) was added as another eye-directed therapy, which in conjunction with OAC became the current standard therapy utilized by many centers in high- and middle-income countries, achieving unprecedented success in eye preservation and completely eliminating the use of EBRT (19, 21–24).

Despite the changing paradigm of retinoblastoma treatment from systemic to OAC and IVi chemotherapy, eyes that relapse or that are initially refractory to conventional therapy are still difficult to treat with currently available drug options and most undergo enucleation. Thus, drug discovery in retinoblastoma is of paramount importance. Recent studies have used innovative multi-omics technology to identify deregulated pathways that could be targeted *via* novel treatment strategies in retinoblastoma. Moreover, targets for immunotherapy such as GD2 ganglioside have been under evaluation despite limited translation into the clinic. Other innovative approaches include conditional replicating oncolytic adenovirus targeting the RB1 pathway, which is currently under Phase I evaluation with promising early results.

In the present review we aimed to describe the latest innovations in retinoblastoma treatment, including both preclinical and clinical studies that have led to new local drug delivery routes and the discovery of promising drugs and non-chemotherapy strategies.

## Local Chemotherapy Administration

In the 1950s, systemic triethylenemelamine was used in patients with retinoblastoma to reduce the dose of EBRT, but was abandoned due to severe side effects (fever with neutropenia was then a life-threatening condition). In the 1970s, systemic chemotherapy was reintroduced for retinoblastoma using vincristine, cyclophosphamide, cisplatin, and doxorubicin based on clinical responses in metastatic patients (25, 26). It was not until the mid-1990s that systemic chemotherapy became widely adopted based on work in England and the US showing the efficacy of carboplatin for intraocular disease (27). Drug selection was mainly on an empirical basis. During the last two decades, etoposide for systemic chemotherapy and both topotecan and melphalan, and also etoposide and vincristine, have been incorporated in the clinic.

After intravenous (i.v) administration, the entire drug dose enters the systemic circulation but only a small fraction becomes available at the ocular tumor after metabolism in major organs and penetration through the blood-ocular barrier. For instance, vitreous topotecan drug exposure was only one-third of the systemic exposure after i.v infusion in rats, whereas etoposide vitreous concentrations barely attained 10% of the systemic exposure (28). Although clinical responses were universal and often dramatic with these drugs, tumors regrew if they could not be controlled with additional focal treatments. The majority of retinoblastoma eyes worldwide at diagnosis have vitreous and sub-retinal seeding which is rarely controlled by systemic chemotherapy (2, 10). Thus, local drug delivery is preferred to increase drug exposure in the eye while limiting the amount that reaches the bloodstream, which reduces systemic toxicity and risk of second malignancies being particularly important for children with germline mutations (29, 30).

During the last decade, rates of globe salvage have dramatically improved, even in eyes with advanced retinoblastoma, as a result of routine local chemotherapy administration. For example, at the Memorial Sloan Kettering Cancer Center (MSKCC) in New York, the enucleation rate dropped from 95% to less than 10% in a ten-year period (18). This success is most probably related to an increase in the bioavailability of the drugs in tumor tissues.

### Ophthalmic Artery Chemosurgery

Intra-arterial delivery of chemotherapy was first achieved 70 years ago by Reese, who delivered a nitrogen mustard derivative to the eye by puncturing the internal carotid artery and injecting the drug into that artery. Although responses were dramatic, tumors regrew; he added EBRT to cure those patients. Japanese investigators then discovered that melphalan appeared to be the most effective chemotherapy against retinoblastoma and began delivering intra-arterially (31). They designed a microcatheter similar to a miniature Foley catheter, which was inserted in the femoral artery and passed through the body until just above the orifice of the ophthalmic artery. After occluding the internal carotid with the balloon catheter, melphalan was injected. The success rate was high and long-term follow-up showed no adverse consequences from the technique, drug, or X-ray exposure during the procedure (32). These patients also received combinations of EBRT, IVi melphalan, and hyperthermia, making it difficult to discern the true contribution of intra-arterial chemotherapy. Despite having experience for more than 30 years with over 400 eyes, the technique was only performed in Japan. The modern era for OAC began following establishment of the technique by Abramson and Gobin at MSK in 2006 (15, 33). Since then, it has been adopted as primary and salvage therapy for both unilateral and bilateral retinoblastoma worldwide and is performed in more than 45 countries. Every series published to date, from the US, China, India, Argentina, Italy, Switzerland, Malaysia, and others, has concluded that the technique is better, safer, and more effective (33–39).

Key outcomes of the widespread adoption of OAC include:

1. Eliminating the use of EBRT, which has decreased the incidence of second, non-ocular tumors and thereby improved long-term survival (2).
2. Eliminating the use of systemic multiagent chemotherapy completely in many centers. This eliminates the need for a port, which requires prophylactic antibiotics to prevent infections, and also for transfusions to treat associated neutropenia. Also, it prevents chemotherapy-induced hair loss and permanent hearing loss (caused by carboplatin). With OAC, fever/neutropenia requiring transfusions develops in <1% of all patients. Associated benefits include:
  - Likely eliminating the development of secondary leukemia in retinoblastoma patients; to date no cases have been reported worldwide (30).
  - Reducing expense in most settings by avoiding the costs of systemic chemotherapy: ports, antibiotics, hospitalization, and treatment for febrile neutropenia such as transfusions (33).
  - Reduced impact on immune function, allowing patients to continue to receive routine vaccinations (40).
  - Avoiding associated hindrance of patient growth (height and weight (41)).
3. Drastically increasing the rate of eye salvage. At MSKCC, this rate has increased from 5 to 95% (42).
4. Enabling salvage of eyes that have failed multiagent systemic chemotherapy (13, 18).
5. Reducing the time from initiating to completing therapy (2).
6. Successful treatment of choroidal invasion, orbital retinoblastoma and optic nerve invasion in select cases (43, 44).
7. Enabling cure of eyes with vitreous and or subretinal seeding which was nearly impossible with systemic chemotherapy and usually failed with external beam radiation.
8. Avoidance of increased risk of orbital disease or second cancers (45).
9. Allowing almost 25% of all eyes with retinal detachment and extinguished ERGs to regain more than 25 μV of activity (46).

The aim in developing OAC was to deliver the drug directly into the artery that irrigates the ocular tissues to increase local bioavailability while minimizing systemic exposure. An initial proof-of-concept study in a swine model reported that topotecan vitreous exposure was almost 30 times higher than that in the bloodstream (47). Moreover, vitreous levels exceeded the drug concentration needed to cause a 50% decrease in *in vitro* tumor cell proliferation (IC_50_) for a period of time greatly exceeding that needed to exert cytotoxicity. A later similar study showed that the OAC route selectively delivers chemotherapy to the eye: topotecan concentrations in the vitreous and retina of non-tumor bearing pigs were 243 and 146 times higher after OAC than after IV, respectively, while systemic exposure was comparable between the two routes (48). The favorable disposition of topotecan in ocular tissues after OAC would have predicted successful tumor control; however, this has not been clinically observed. Failure to achieve adequate tumor control despite vitreous levels above the IC_50_, considered a surrogate of active pharmacological threshold, may be related to the administration schedule. Because topotecan exerts its cytotoxic activity by inhibiting the nuclear enzyme topoisomerase I, resulting in double-strand breaks during cell replication, it acts on cells in the S-phase and subsequent doses are required to target quiescent tumor cells that enter cell replication. Thus, protracted schedules have been associated with greater antitumor effects compared to intermittent, higher-dose schedules (49, 50). However, repeated daily OAC to deliver topotecan is impractical, limiting its efficacy *via* this delivery route.

OAC infusion was also shown to allow more selective ocular delivery of the alkylating agent melphalan; concentrations in the retina and vitreous were 12 times and 26 times higher, respectively, after OAC than after IV infusion in non-tumor-bearing rabbits (51). Interestingly, as these values are much lower than those for topotecan, and considering that vitreous-to-plasma exposure of melphalan also in pigs is 10-times less than that for topotecan, melphalan appears to display a lower capacity to penetrate or be retained in the vitreous and retina than topotecan (52, 53). This limited penetration and/or retention of melphalan in the vitreous after OAC was evident in a pharmacokinetic study in pigs showing that the drug barely attains cytotoxic levels in the vitreous (53).

Despite restricted melphalan vitreal penetration in non-tumor bearing models, with the caveat of potential differences in drug disposition between eyes filled with tumor and healthy eyes, melphalan displays a marked antitumor effect after OAC that is not observed after topotecan. Nonetheless, the higher ocular exposure to chemotherapy attained after OAC delivery than after IV infusion is the reasonable explanation for a better tumor control in eyes with retinoblastoma. This hypothesis was confirmed in tumor-bearing rabbits treated with a high dose of OAC melphalan (1.2 mg/kg), which showed substantial antitumor effects, in contrast to a lack of tumor cell killing in eyes treated with the same IV dose of melphalan or with the standard combination of carboplatin, vincristine, and etoposide that is still widely used in clinical practice (51). Nonetheless, these results may be biased by the use of a single dose of melphalan almost 3 times higher than that used for patients with retinoblastoma. Melphalan cytotoxicity is schedule and dose-dependent and these factors may have an impact on the drug efficacy and toxicity.

Other results that support the penetration of melphalan after OAC in ocular tissues were obtained in pigs in which melphalan accumulated in the retinal pigment epithelium (RPE)-choroid, probably due to its affinity to melanin and perhaps explaining the efficacy against subretinal seeds, and the choroidal toxicity associated with the drug in the clinic (53).

Although OAC primarily delivers drug locally, because the drug is injected into an artery, detectable concentrations in the bloodstream are expected as shown in animal models (47, 51, 53). After OAC of melphalan to rabbits, drug concentrations in the contralateral eye were 50–80 times lower than in the treated eye, but similar to those in plasma (51). Importantly, a clinical pharmacokinetic study in patients receiving single-agent melphalan by OAC reported that melphalan dose-normalized systemic exposure was 165 ng ∗ h/ml/mg. As it is well described for IV infusions, melphalan systemic exposure after OAC is proportional to the dose (between 3 and 7 mg), and a threshold of 0.48 mg/kg is associated with an increased probability of developing severe neutropenia (53, 54). In a subsequent study, the pharmacokinetics of melphalan was unaltered by concomitant administration of topotecan. Consistent with similar systemic exposure to melphalan, the incidence of grade III/IV neutropenia (12%) was comparable to that obtained in patients receiving OAC of single-agent melphalan at doses less than 0.5 mg/kg, and no patient had fever or needed transfusions or hospitalization (55). In addition, topotecan systemic exposure was far less than that previously reported to be associated with severe neutropenia (56).

Recent work showed that OAC is also a selective route for carboplatin delivery to the retina, resulting in an almost 4-fold higher exposure in this tissue compared to that in plasma (51). Nonetheless, the blood-retinal barrier may hinder free drug penetration, as carboplatin exposure in the vitreous was almost 3 times lower than in the retina. Despite potential ocular barriers limiting free diffusion, OAC led to 123 and 131 times higher exposure in the retina and vitreous, respectively, compared with those following IV infusion of the same dose (51).

The most common toxicity of OAC results from the high dose of chemotherapy in the eye, namely choroidal vascular toxicity, which arises in approximately 3–5% of patients (57). Central retinal artery occlusion is also possible after OAC, but has been reported in isolated cases and is clearly related to the experience of the treating team (58). Other side effects such as eyelid edema are seen in 10–15% of infusions, but do not give rise to permanent sequelae (42). Some patients present hypotension and bronchospasm during the anesthetic procedure, which may occasionally lead to respiratory arrest (59). Stroke has not been seen in most large series, but single cases have been reported after OAC (60).

The optic nerve is a crucial route of tumor cell dissemination to the CNS in eyes with advanced disease, and thus patients with optic nerve tumor involvement would benefit from high local exposure to chemotherapy (61). Because the ophthalmic artery supplies blood to the intraorbital segment of the optic nerve, in a porcine model, OAC administration of topotecan led to drug concentrations that were 80 times higher in the proximal and distal portions of the optic nerve than after IV infusion, with concentrations greatly exceeding the topotecan IC_50_ (48).

These concepts were translated to the treatment of a patient with a massive orbital and chiasmatic mass with positive CSF cytology. This patient received high-dose OAC targeting the optic nerve and chiasm, along with intrathecal topotecan for leptomeningeal dissemination (44). Histologically confirmed complete response was achieved after three cycles of this treatment. Orbital retinoblastoma has also been treated with OAC in a limited number of patients, either as monotherapy after extraocular relapse or in combination with systemic chemotherapy to avoid the use of orbital EBRT (62, 63).

Typically, massive choroidal invasion is detected upon pathological examination of the enucleated eye and has been associated with up to 4% risk of extraocular relapse (64). OAC has also been recently used for the treatment of patients in whom choroidal invasion was detectable during ocular examination (43). In these patients, tumor response included the choroidal invasion and eyes were preserved without extraocular relapse. Though these are encouraging results, more data are needed to confirm the safety of this therapy.

### Intravitreal Injection

Vitreous seeding may occur in eyes with endophytic features or after conservative therapy. Until the advent of OAC in combination with IVi, vitreous seeding was extremely difficult to control with systemic chemotherapy or EBRT, and thus most of those eyes were enucleated. Modifying IVi to enhance safety by injecting the drug in a tumor-free pars plana site using an anti-reflux technique made it possible to safely administer chemotherapy and, more recently, biological agents (21, 65, 66). A worldwide survey of thousands of cases revealed no extraocular spread after IVi (65). Similarly, using a sensitive method for the quantification of RNA from photoreceptors collected *via* sterile filter paper pieces placed on the site of injection after IVi, no tumor cell reflux was detected after IVi chemotherapy (61, 67).

Melphalan and topotecan have been the most commonly used drugs which may be injected every 7–21 days; the number of injections varies depending on response and toxicity (23, 68–70). In general, response of vitreous seeds to IVi injections varies from 85 to 100% efficacy (23, 69). Response to IVi chemotherapy, including time to regression, is associated with seed classification according to appearance on indirect ophthalmoscopy as clouds, dust, or spheres (69, 71). For instance, spheres regress in a shorter amount of time and receive less cumulative drug compared with clouds.

Retinal toxicity has been reported with IVi melphalan: specifically, each 30 µg of melphalan is associated with an approximately 5 µV decrement in retinal function as evaluated by electroretinogram (68). Because melphalan physically associates with melanin, eyes with deeper inherent pigmentation appear to develop more retinal toxicity following IVi, i.e., worse electroretinogram recordings and more apparent fundus abnormalities (23, 68, 70), due to uptake into the RPE. IVi topotecan does not appear to confer the same retinal toxicity (72), although its clinical utility as a single agent through IVi is still being investigated. There are only limited data on the efficacy and toxicity of IVi carboplatin (73).

IVi injection is ideal for targeting vitreous disease, as this route avoids losing active drug due to restricted penetration through the ocular barriers and systemic metabolism. IVi injection of only 15 μg of melphalan, equivalent to a human dose of between 30 and 35 μg, resulted in vitreous concentrations above the IC_50_ for several hours, consistent with IVi administration allowing superior control of vitreous seeds in patients (74). Importantly, melphalan was undetectable in plasma, probably explaining the lack of hematologic toxicity associated with IVi chemotherapy. Although beneficial for controlling vitreous seeds, high drug concentrations in the vitreous were associated with morphological and functional retinal changes in white and pigmented rabbits and also a reduction in the ERG response in patients (23, 68, 75–78). As mentioned earlier, this toxicity likely results from the melanin-binding capacity of melphalan and resulting retention in the RPE (53, 68, 79).

Some practical considerations that hinder the use of melphalan include its spontaneous hydrolysis; significant activity is lost after 1 h of reconstitution of the commercial vial. Moreover, as the commercial vial contains 50 mg of melphalan but doses for IVi delivered in 0.1 ml are typically between 20 and 30 μg, about 94% of the drug would be wasted at every single procedure. To avoid this unnecessary cost, pharmacists have shown that storing reconstituted melphalan solution (300 ng/ml) in prefilled syringes at −20°C maintains activity (80). In recent years, a new propylene glycol-free injectable melphalan formulation has shown improved drug stability compared to the original product (81) without affecting the frequency of toxicity and ocular survival in patients receiving IVi melphalan (76).

A safer cytotoxic agent for IVi is the topoisomerase inhibitor topotecan, as shown in preclinical models and in human eyes of the single agent at doses up to 30 μg or in combination with melphalan (72, 75, 82–84). As previously discussed, protracted schemes of topotecan and thus, high and prolonged vitreous exposure would be desirable to leverage antitumor efficacy without the need for further IVi injections (50, 85). Three weekly doses of 15 μg or 30 μg topotecan were reported as nontoxic to the rabbit eye (75). In our experience repeated doses of up to 50 μg of topotecan in rabbits (equivalent to a 100 μg human dose) can be intravitreally injected without morphological or functional retinal changes (86).

Lastly, an advantage of using topotecan is that institutions with limited resources may opt to use a solution of 300ng/mL in prefilled syringes at room temperature for 24 h and frozen at −20°C as they have proven to be stable for almost 6 months (80).

### Intracameral Chemotherapy

Invasion into the anterior segment was traditionally considered an absolute indication for eye enucleation, based on the possible increased risk of systemic dissemination *via* the heavily vascularized structures of that part of the eye. However, many patients respond to repeated intra-cameral injection of chemotherapy, usually in combination with IVi chemotherapy, and many such treated eyes could be saved. However, because of the rarity of anterior segment invasion, experience is still based on a limited number of cases. As in the case of IVi chemotherapy, melphalan and more recently topotecan have been used for intracameral injection (2, 87).

### Periocular

Periocular injection of chemotherapy has the advantage of circumventing the blood-retinal barrier, reaching the tumor through transscleral passage without the need to puncture the globe. Nonetheless, orbital and choroidal blood flow, tissue binding, and tight junctions between RPE cells hinder free drug transscleral diffusion.

Carboplatin has been the most intensively investigated drug and although it resulted in a substantial increase in vitreous concentrations in preclinical models, it was abandoned by most groups because of its local and profound hematopoietic toxicity if given in combination with systemic topotecan (88–90). Due to the physicochemical properties of topotecan, it diffuses across the sclera less efficiently than carboplatin and therefore reaches the vitreous after distribution from the bloodstream, as shown in non-tumor-bearing rabbits (91). Nonetheless, a phase I study was opened to evaluate a novel sustained-release episcleral plaque loaded with topotecan called “Chemoplaque” (92).

### Intrathecal

Patients with leptomeningeal retinoblastoma are seldom cured with current therapy. In these cases, intrathecal (IT) or intraventricular (IVt) administration of chemotherapy may provide a means of attaining pharmacologically active levels in the CSF. These routes ensure direct delivery of chemotherapy to the CSF and thus are particularly advantageous for drugs whose free penetration is restricted by the blood–brain barrier.

Intrathecal methotrexate and cytarabine were used to treat retinoblastoma in the 1980s but *in vitro* results showed low or modest cyototoxicity in retinoblastoma cell lines (31, 93). More recently, IT/IVt topotecan has been introduced. Moreover, administration of topotecan is feasible at a maximum dose of 0.4 mg with manageable toxicity and clinical responses in patients with leukemia (94). In retinoblastoma, IVt topotecan and cytarabine in combination with IV or OAC chemotherapy have been effective in a few cases (95). A current Latin American GALOP group-led study is testing the use of IT topotecan in the adjuvant setting (96, 97); more such studies are necessary to determine the role of IT topotecan in retinoblastoma. Other putative candidates for intrathecal use include romidepsin, etoposide, and teniposide (98).

## Drug Discovery in Retinoblastoma

New drugs effective for both intraocular and disseminated retinoblastoma are urgently needed; these may include novel drugs and repurposed agents, i.e., those already used in the clinic for other cancers or diseases but still without an indication for retinoblastoma. Assessing response to treatment in the preclinical setting is a challenge due to the limited availability of models that recapitulate clinical observations. Similarly, assessing clinical response in the setting of intraocular disease is difficult because of the lack of consensus for response criteria. The RECIST response criteria were recently proposed to address this issue, but they require MRI, which is expensive and unable to assess small tumors, and ultrasound, the use of which is not standardized worldwide and not reproducible in retinoblastoma (99). Current approaches to developing new treatments include drug discovery using innovative technology to identify deregulated pathways in specific tumors, against which targeted agents may be designed, and drug repurposing, a faster and cheaper strategy.

**Figure 1:**
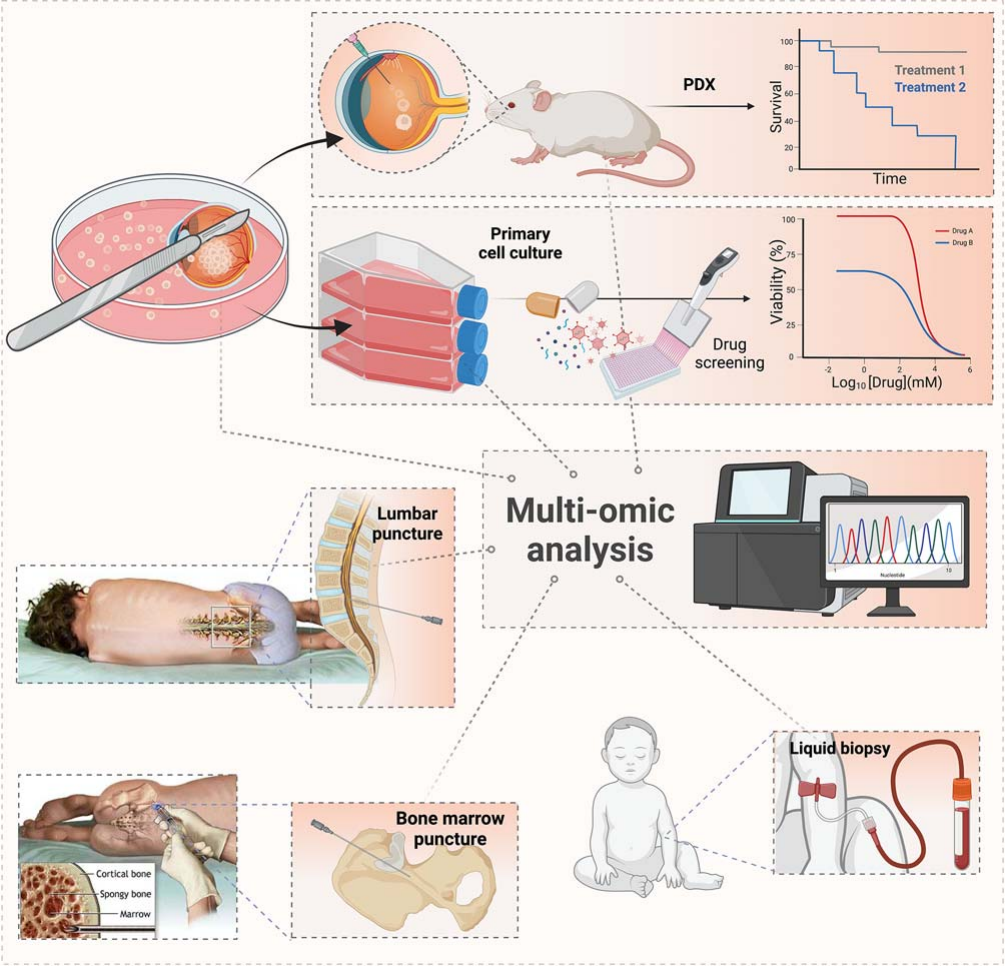
Workflow for tumor sampling and genomic and pharmacological characterization. Samples obtained from enucleated eyes with retinoblastoma, cerebrospinal fluid, and/or bone marrow are subjected to multi-omic analysis to identify deregulated pathways that may be subjected to targeted agents. Also, these samples may be used to establish tumor cell lines and patient-derived xenografts in immunocompromised animals to evaluate tumor invasiveness and pharmacological sensitivity to novel agents, repositioned drugs, or combination therapies.

The discovery of new drugs with potential for translation to the retinoblastoma clinic should be rationally designed to prioritize the most promising candidates. The fundamental starting point in the selection process is comprehensive pharmacological characterization to determine the capacity of each agent to induce selective cytotoxicity in tumor cells using commercial or primary cell lines derived from intraocular tumors or sites of dissemination (100–105) (**Figure 1**). Such evaluation may consist of high-throughput screens (HTS) in which large libraries of hundreds of compounds are rapidly tested, though few HTS screens have been reported, likely due to challenges in establishing and maintaining retinoblastoma cell lines. Preferably several cell lines should be tested to account for the diversity in drug response observed in patients. Animal models bearing tumors xenografted from intraocular or metastatic patient retinoblastomas could provide a strategic tool to evaluate drug performance before translation to humans (106, 107) (**Figure 1**).

One HTS study identified cardenolides as active against retinoblastoma (108). Based on this finding, one patient was reported cured from recurrent intraocular disease after OAC delivery of the cardenolide digoxin, but a cataract developed as a consequence (109). Nonetheless, OAC may not be the optimal route for digoxin due to low intravitreal levels and systemic exposure related to cardiotoxicity; IVi may be preferable. In rabbits, IVi of digoxin resulted in levels well above the IC_50_ and plasma concentrations far below the concentrations related to cardiac toxicity in humans; however, the injected dose was toxic to the retina (110, 111).

Subsequently, almost 200 compounds were found to be cytotoxic to primary cell lines derived from intraocular and CSF-disseminated retinoblastomas, including drugs already used in pediatric oncology and new hits in preliminary phases of drug development (98). A selection process for prioritizing drugs for subsequent studies before clinical translation based on their pharmacokinetics, biopharmaceutical properties, safety profiles, and the intent of treatment was proposed. Further, FDA-approved agents that have been investigated in Phase I/II studies in pediatrics or clinically used in this age group may be more rapidly translated into the clinic as repurposed drugs. However, an alternative option would be to identify tumor targets and then select or develop direct treatments. In all cases, one of the first steps in the decision process is to consider the properties of a given drug (solubility, metabolism, and ability to cross the blood-ocular or blood–brain barrier) in light of potential delivery routes and corresponding clinical uses (OAC and IVi for intraocular disease and IT/IVt to target tumor cells that have spread to the CNS) (**Table 1**).

**Table 1:** Characteristics for drug selection according to the route of administration.

Route	Characteristics						
	Injectable aqueous solution	Not a prodrug	Devoid of vesicant effect	Penetrate the BBB/BOB	Devoid of ocular & neurological toxicity	Devoid of systemic severe toxicity
**Local treatment**							
**Intravitreal**	Required	Required	Required	Not required	Required	Not required
**Intrathecal/intraventricular**	Required	Required	Required	Not required	Required	Not required
**OAC**	Required	Required	Required	Required	Required	Required
**Systemic treatment**						
**IV**	Required	Not required	Not required	Required	Required	Required
**Oral**	Not required	Not required	Not required	Required	Required	Required

BOB, blood-ocular barrier; BBB, brain–blood barrier.

**Figure 2:**
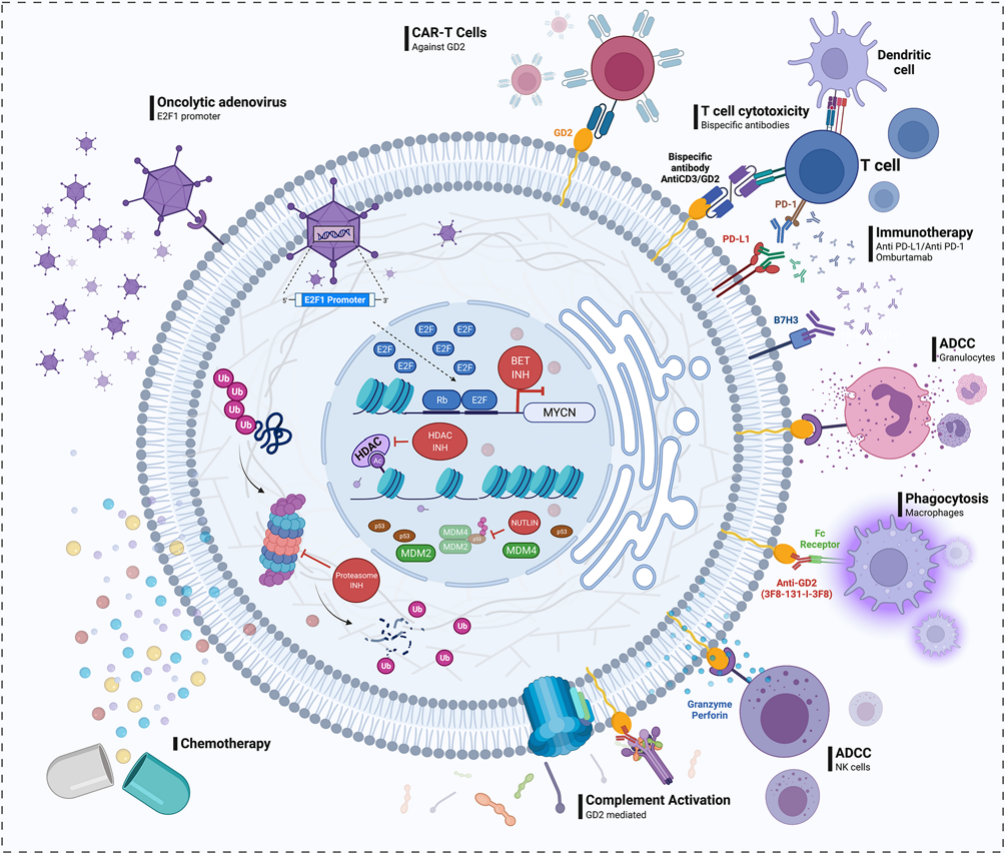
Drug discovery in retinoblastoma. Left: Pathways shown to be deregulated in retinoblastoma and drugs targeting them that may represent future candidates for translation into the clinic. These include agents that disrupt the interaction between MDM2/MDM4 and p53 (e.g., nutlin); proteasome, and histone deacetylase (HDAC) inhibitors; bromodomain extra-terminal (BET) proteins inhibitors, which may be selectively active against tumors with *MYCN* amplification; and a genetically modified adenovirus that selectively replicates in cells with high levels of free E2F transcription factor resulting from a dysfunctional RB1 pathway. Right: Proposed immunotherapy approaches in retinoblastoma, namely, anti-GD2 and anti-NcGM3 monoclonal antibodies, anti-GD2CART cells, and inhibitors of immune checkpoints as PD-1/PD-L1 and B7H3 (omburtamab). In the presence of anti-GD2 monoclonal antibodies, retinoblastoma cells may be targeted by the immune system, as described in neuroblastoma, *via* a response including granulocyte- and natural killer-mediated antibody-dependent cellular cytotoxicity (ADCC) and complement- and macrophage-mediated cytotoxicity.

According to this approach, promising agents in early phases of drug development, namely, inhibitors of Bcl-2 proteins, the proteasome, bromodomain and extra-terminal motif proteins (BET), NF-κB, histone deacetylase, kinesin spindle protein, STAT3, and survivin, were identified (**Figure 2**). Similarly, functional genomics and transcriptomic analysis of retinoblastoma tumors showed the DNA repair proteins RAD51 and BRCA1 to be deregulated, leading to evaluation of the combination of topotecan, a DNA-damaging drug, with a specific small molecule inhibitor of RAD51, which resulted in synergistic *in vitro* and *in vivo* antitumor effects (112). A triple combination with the BCL-2 anti-apoptotic inhibitor navitoclax was proposed to lower the dose of the synergistic combination and avoid tumor cell resistance.

One member of the ubiquitin-proteasome system shown to be critical for *RB1*^−/−^ retinoblastoma is the cullin-RING E3 ligase Skp2 (113, 114). Translation of these results into a novel pharmacological development is limited by the low potency of Skp2 inhibitors. An alternative approach to disrupt ubiquitylation is to inhibit activation of the cullin component using pevonedistat, which is in more advanced stages of drug development in models of neuroblastoma (115–117). IVi pevonedistat, selected as the delivery route because of the low permeability of the drug across the blood–brain barrier, resulted in antitumor effects in *in vitro* and *in vivo* preclinical models including *MYCN* amplified retinoblastomas without evidence of ocular toxicity.

A recent analysis of single-cell sequencing data identified molecular pathways that are deregulated in retinoblastoma and could be targeted as novel treatment strategies (118). Among the most deregulated was the MDM2/MDM4-p53 pathway (119). Although the gene *TP53* is almost always unaltered in retinoblastoma, the negative regulator MDM2 and its homolog MDM4 may be overexpressed, blocking the transcriptional activity and stimulating degradation of the p53 protein (120). Therefore, novel drugs such as nutlin-3, which inhibits the interaction between MDM2 and MDM4 with p53, have been pursued as a strategy to restore p53-mediated apoptosis in tumor cells (121, 122) (**Figure 2**). Nonetheless, contradictory results regarding the incidence of MDM2/MDM4 overexpression in patients with retinoblastoma, along with the toxicity of nutlin-3a and poor bioavailability in ocular tissues, prevented its translation into the clinic despite promising results in genetically engineered and xenograft models (120, 122, 123). New classes of small molecule MDM2/MDM4 inhibitors are being evaluated in Phase I/II studies in combination with broad-spectrum and targeted therapies; one of these is a trial in pediatric cancer including retinoblastoma (121, 124). Another option is targeting another p53 negative regulator, HDM2, a small molecule inhibitor which have been shown to cause cell death in retinoblastoma cells (125).

Other pathways that were among the most significantly deregulated in the above single-cell sequencing study included *MYC* and GABA receptor signaling, both activated, prompting further investigation of inhibitors of *MYC* signaling, such as MYC/MAX interaction inhibitors and BET inhibitors, and repurposing of calcium and potassium channel blockers (118) (**Figure 2**). Expression of members of the retinoid X receptor family was also found to be altered, supporting vitamin D and retinoic acid, the ligands of these receptor, as candidate retinoblastoma therapeutics. Finally, the NF-κB, IGF1R, and STAT3 pathways were also deregulated; agents that target these pathways have already been proposed (98).

Epigenetic alterations have also been investigated as alternative targetable pathways for retinoblastoma treatment. A multi-omic study showed that epigenetic changes, including upregulation of *SYK*, cooperate with *RB1* loss to support retinoblastoma tumor progression (126). The *SYK* inhibitor BAY-61-3606 led to apoptosis in retinoblastoma cell lines and resulted in antitumor effects after subconjunctival administration in orthotopic xenografts. In contrast, the *SYK* inhibitor fostamatinib, an orally available prodrug that has recently been approved for the treatment of adult patients with chronic immune thrombocytopenia, and the active metabolite R406, had no antitumor effects in tumor-bearing animals when administered *via* several local routes and in multiple formulations (127–129). This lack of antitumor activity calls into question the clinical relevance of targeting epigenetic deregulation of *SYK*.

Continued efforts to identify alterations in signaling and regulatory pathways driving retinoblastoma development and progression promises to contribute to the development of novel targeted agents and bring new combination therapies to the clinic.

### Gangliosides and Other Targets for Immunotherapy

Retinoblastoma widely expresses the ganglioside GD2 even in pre-treated chemo-refractory cases with *MYCN* amplification (105, 130, 131) (**Figure 2**), suggesting that anti-GD2 monoclonal antibodies may be effective. These antibodies are approved for the treatment of neuroblastoma (132, 133), but there is little clinical experience with their use in retinoblastoma and its niche therapeutic effect (if any) needs to be ascertained.

The major toxicity of anti-GD2 antibodies is related to peripheral nerve toxicity, limiting their use as conservative therapy for retinoblastoma and for the treatment of extraocular disease as the major site of failure in these cases is the CNS. Nonetheless, the radio conjugated anti-GD2 murine antibody ^131^I-3F8 was shown to be well-tolerated in a Phase I study when delivered from an Ommaya reservoir for control of CNS metastasis (134). Other gangliosides such as NcGM3 are also highly expressed in retinoblastoma and may represent potential immunotherapy targets, as shown in a child with trilateral retinoblastoma who was treated with an anti-idiotypic vaccine targeting NcGM3 (135).

GD2 along with the neural cell adhesion glycoprotein CD171, which is also highly expressed on the surface of retinoblastoma cells, is also a target for recently designed CART cells. Sequential administration of these two types of CART cells was cytotoxic to a panel of retinoblastoma cell lines (136). Subsequently, others developed local immunotherapy formulations also based on anti-GD2 CART cells, including an injectable hydrogel to prolong their localization at the site of injection. After IVi administration, the formulation resulted in complete tumor response and no recurrence or ocular toxicity in orthotopic xenograft models (137, 138) (**Figure 2**).

Though the eye is traditionally considered an “immune-privileged” organ (139), recent transcriptomic data on initially enucleated eyes revealed an immune signature specific to a retinoblastoma subtype characterized by a putative “late cone” precursor (subtype 1 retinoblastoma) (140). These cases, compared with those originating from earlier cone precursors which may show an immunosuppressive signature as in other neuroectodermal tumors (subtype II retinoblastoma), have relatively high infiltration of immune cells in the tumor microenvironment. Moreover, PD-1 and PD-L1 expression were observed in 20–40% of cases, depending on whether the eye was primarily or secondarily enucleated (141–143), while CTLA-4 expression followed a similar pattern. Some retinoblastomas also express B7H3 (144), another immune checkpoint target for which omburtamab has been developed for clinical use as radio-immunotherapy to treat other neural and mesenchymal tumors (**Figure 2**). These findings support the investigation of immune checkpoint inhibitors for retinoblastoma, but such studies have not yet been undertaken.

### Gene Therapy and Oncolytic Virus Treatments

The eye presents a favorable environment for intraocular injection of therapeutic viral vectors. IVi administration of an adenoviral vector carrying a suicidal gene followed by ganciclovir therapy was tested in a Phase I study, which reported that one of eight patients treated achieved tumor control (145). However, the advent of effective IVi chemotherapy limited its subsequent development. Other groups carried out preclinical development of conditional replicating oncolytic adenovirus. One of these vectors was clinically translated as VCN-01, a genetically modified adenovirus designed to selectively replicate in cells with high levels of free E2F-1, which results from RB1 pathway dysfunction (66, 146). Preclinical studies showed high antitumor activity in a set of patient-derived cell lines and xenografts with little or no dissemination to other organs and no replication in healthy retinas after IVi. The experience was translated to the clinic and a Phase I study is currently open with initial results revealing antitumor activity in one of the treated patients (66).

## Noninvasive Diagnosis of Tumor Dissemination

Recent studies suggest that biological subtypes of retinoblastoma may differ in their ability to metastasize. The more aggressive genetic subtype 2 is more prevalent among patients with unilateral disease, who may be at higher risk of tumor dissemination (140). However, our capacity to noninvasively identify patients at higher risk for developing metastatic disease or those with *MYCN*-amplified tumors who are not candidates for eye salvage is still limited (147). A promising method of noninvasive tumor monitoring is the measurement of circulating tumor DNA (ctDNA) in liquid biopsy samples. Liquid biopsy studies have shown that patients who subsequently developed metastatic relapse had higher levels of ctDNA (148, 149). In these patients, ctDNA levels could indicate higher tumor burden, and continued ctDNA detection after enucleation would predict impending extraocular relapse. Another major site of tumor dissemination is the CSF, but yet it has not been sampled for liquid biopsy study in retinoblastoma.

## Discussion

Retinoblastoma has been selected as a priority tumor by the WHO for its Global Initiative for Childhood Cancer. Despite being highly curable at early stages, it may be fatal if left untreated. Eye-globe salvage treatments have substantially evolved over the last few decades, making intraocular retinoblastoma the most curable of all pediatric cancers in high-income countries. The development of local drug delivery methods that maximize chemotherapy exposure in the retinal, subretinal, and vitreous spaces, namely OAC and a safety-enhanced technique for IVi injection, have resulted in an unprecedented rate of eye globe and vision preservation. Importantly, these new local treatments result in very high concentrations of chemotherapy in the retina and optic nerve as shown in preclinical models, preventing dissemination to the CNS. So far, after more than a decade of continuous use at major clinical centers around the world and after more than 200 articles published in the field, IVi and OAC have been proven safe without increasing the risk of metastatic dissemination. By eliminating the use of EBRT and systemic chemotherapy, these treatments have improved long-term survival by reducing the incidence of treatment-associated severe toxicities, the risk of secondary malignancies, and related deaths.

In contrast to this exceptional improvement in treatment outcomes, children with disseminated retinoblastoma have few therapeutic options, generally limited to high-dose chemotherapy, stem cell transplant, and local radiotherapy. Even worse is the scenario for patients with metastasis in the CNS, as these patients seldom survive even with intensive therapies. Thus, newer treatments and improved methods of targeting drug delivery to the CNS may improve outcomes. Examples of CNS-targeted routes include IT and IVt injection, which ensure direct delivery of chemotherapy to the CSF, circumventing the blood–brain barrier. OAC may also be useful in patients with orbital retinoblastoma with massive optic nerve and chiasmatic tumor involvement because of maximal local exposure to chemotherapy as shown in animal models. Future clinical assessments are necessary to determine the role of local chemotherapy delivery in disseminated retinoblastoma.

In all cases of intraocular and extraocular disease, there is a need for new therapies that are more effective and carry less risk of toxicity. New treatment modalities, namely, targeted therapies, immunotherapy, and oncolytic viruses are emerging as possible non-chemotherapeutic options. These novel treatments may further reduce the use of cytotoxic agents, potentially leading to even higher ocular preservation rates, reduced toxicities, and prevention of tumor dissemination.

Identifying high-risk features associated with tumor progression and metastasis by histopathological analysis of the enucleated eye is critical for selecting appropriate management. These approaches may soon be supplemented by ctDNA analysis, which may be an early and noninvasive prognostic biomarker of treatment response and risk of occult extraocular dissemination. In addition, ctDNA may be helpful in noninvasive genomic profiling, especially to identify patients with the subtype 2 molecular signature who have an increased risk of extraocular relapse.

In summary, retinoblastoma treatment has evolved over the last century, resulting in a striking change in the treatment paradigm of this ocular tumor. Advances in the knowledge of its tumor biology and drug response and the development of new routes of drug delivery promise to lead to additional new, more effective, and less toxic therapies in retinoblastoma.

## Author Contributions

Conceptualization, initial idea: PS, GC, and DHA. Manuscript preparation: PS, JHF, MBC, GC, AMC, and DHA. Supervision and manuscript review: PS, GC, and DHA. All authors listed have made a substantial, direct, and intellectual contribution to the work and approved it for publication.

## Funding

This work was funded by the Fund for Ophthalmic Knowledge, NY, USA. The funders had no role in the decision to publish or preparation of the manuscript.

## Conflict of Interest

GC has received speaker honoraria from Ymabs, Tecnopharma, and Bayer. 

The remaining authors declare that the research was conducted in the absence of any commercial or financial relationships that could be construed as a potential conflict of interest.

## Publisher’s Note

All claims expressed in this article are solely those of the authors and do not necessarily represent those of their affiliated organizations, or those of the publisher, the editors and the reviewers. Any product that may be evaluated in this article, or claim that may be made by its manufacturer, is not guaranteed or endorsed by the publisher.
